# Variation of Morphological, Agronomic and Chemical Composition Traits of Local Hazelnuts Collected in Northern Spain

**DOI:** 10.3389/fpls.2021.659510

**Published:** 2021-06-15

**Authors:** Ana Campa Negrillo, Roberto Rodríguez Madrera, Belén Suárez Valles, Juan Jose Ferreira

**Affiliations:** ^1^Plant Genetic Group, Regional Service for Agrofood Research and Development (SERIDA), Villaviciosa, Spain; ^2^Roberto Rodríguez Madrera, Food Technology Area, Regional Service for Agrofood Research and Development (SERIDA), Villaviciosa, Spain

**Keywords:** field collection, local diversity, phenotyping, oil composition, *Corylus avellana* L.

## Abstract

Hazelnut is a traditional crop in northern Spain, where it grows wild as well as being cultivated. A field collection of 41 local and 17 non-local accessions, including 15 well-known cultivars, was established at SERIDA in Villaviciosa, Spain. Here, phenotypic variation was documented for phenological and morphological traits and chemical composition. A large degree of variation for most morphological and phenological traits, except nut maturity date, was revealed. Estimates of broad-sense heritability were high (>0.75) for most of the assessed characters, except for the first male bloom date (0.65), male and female flowering periods (0.40, 0.31), kernel weight (0.69), and kernel percentage (0.33). Local accessions produced smaller nuts and kernels than well-known cultivars but with higher kernel percentage. Limited overlapping between the male and female flowering periods (dychogamy) was observed, except for ‘Forcinas 1’, ‘Forcinas 2’, and ‘Morell’. The local accessions generally exhibited significantly later male and female flowering compared with the reference cultivars. The local materials showed similar nutritional values to those reported previously for hazelnut. Moreover, the local accessions presented average values similar to the non-local accessions for total fat, ash and carbohydrate contents, as well as energy value, but their protein contents were lower. Their oils were rich in functional compounds, such as unsaturated fatty acids (average: 90.1%), tocopherols (514 mg/kg) and squalene (294.3 mg/kg). A hierarchical clustering on principal components analysis grouped the accessions and differentiated eight local accessions from the rest, including the landrace ‘Casina’. This finding provides potential new cultivars, as well as sources of desirable traits, for European hazelnut breeding programs.

## Introduction

European hazelnut (*Corylus avellana* L.) is an important crop worldwide among the cultivated tree nut species. The crop has increased and spread in recent years (FAOSTAT, 2020). Hazelnut kernels are directly consumed fresh or roasted, although most of the product is used by industries, such as the confectionary industry. Hazelnut kernels are mainly known for their high oil content (∼60%), which includes high concentrations of oleic, linoleic and palmitic acids (Serra and Ventura, 1993; Köksal et al., 2006; Müller et al., 2020). Additionally, hazelnut kernels contain appreciable amounts of other macronutrients, such as proteins (∼17%), and micronutrients, such as vitamins B and E, with tocophenol as the most abundant form (Savage and McNeil, 1998; Köksal et al., 2006; Müller et al., 2020). Proteins from hazelnut are rich in essential amino acids, such as arginine and leucine, and non-essential amino acids, such as glutamic and aspartic acids (Köksal et al., 2006). Significant amounts of K, Mn, Ca, and Mg are also found in kernels. Thus, hazelnut is considered a functional food because its consumption is associated with several human health benefits owing to the high concentrations of bioactive compounds, including sterols, tocopherols, phenolic acids and flavonols (Pelvan et al., 2017). In fact, hazelnut consumption has been linked to the prevention of cardiovascular diseases (Orem et al., 2013).

Hazelnut cultivars, whether from breeding programs or local selection, are clonally propagated by layering the suckers. Plant and nut characteristics are criteria in the selection of cultivars when designing orchards. Among these characteristics, phenological traits are of great importance. Hazelnut is a wind-pollinated and monoecious species that has a sporophytic incompatibility system controlled by a single locus with multiple alleles (Mehlenbacher, 1997). Owing to this pollination system, flowering period and genetic compatibility must be taken into consideration when planning orchards (Germain and Sarraquigne, 2004). Nut phenotypes vary among cultivars, and these traits are highly dependent on the genotypes (Bioversity FAO and CIHEAM, 2008; Yao and Mehlenbacher, 2008). Nut phenotypes are also correlated with the fresh or processed uses, because specific characteristics such as kernel shape and size, kernel percentage, flavor, ease of pellicle removal after kernel roasting, hardness, storage life and nut shell color, are valued (Botta et al., 2019).

Hazelnut is a traditional crop in northern Spain where it also grows in wild forms. Cultivated forms are found in small orchards, gardens and hedgerows, while wild forms are found along stream banks or forming small woods in isolated areas. In the past, the hazelnut was an important crop in Asturias, as reported by Fray Toribio de Santo Tomas (1711–1714) (López et al., 2012). In the mid-twentieth century, Álvarez-Requejo (1965) described the landraces ‘Casina’, ‘Quiros’, ‘Espinaredo’ and ‘Amandi’. However, by the end of the twentieth century, hazelnut cultivation had decreased and the local harvest was only consumed by the owners or sold in local markets. To study and preserve the local genetic diversity, local germplasms was investigated in Asturias (northern Spain) over three consecutive years (2003–2005). The local materials formed a specific germplasm group within *C. avellana*, in which germplasm groups from northeast Spain, southern Italy and the Black Sea region had been previously reported (Boccacci et al., 2013). A genetic diversity analysis using ISSR and SSR markers revealed that the collected local materials were strongly related, constituting a group different from the genotypes grown in northeast Spain and Italy. The local materials also included intermediate forms that were probably derived from crosses between cultivated and wild materials (Ferreira et al., 2010; Campa et al., 2011).

The *in situ* characterization showed that the local materials had phenotypically diverse tree and fruit characteristics (Ferreira et al., 2010). However, morphological and phenological descriptors can be modified by environment; therefore, an accurate morphological characterization requires the evaluation of materials in the same environment over several years. This detailed evaluation of local germplasms can reveal new genotypes specifically adapted to local growing conditions. A field collection with 41 local and 17 non-local accessions was established in SERIDA to preserve and study the local genetic diversity of hazelnut. This work describes the observed phenotypic variations in phenological and fruit morphological characteristics, as well as the chemical composition, among hazelnuts maintained in this collection.

## Materials and Methods

### Plant Material

In all, 58 accessions maintained in the SERIDA collection (Villaviciosa, Asturias, Spain; 43°28′31″N–5°25′7″W) were characterized in this work. The field collection had two trees (clones) per accession (8–10 years old). The material included 37 accessions derived from the local germplasm studies carried out during 2003–2005 and, four accessions derived from the work of Álvarez Requejo (1965), ‘Amandi’, ‘Casina’, ‘Espinaredo’ and ‘Quiros’ (all them considered as local accessions). Fifteen well-known cultivars, representing a wide phenotypic diversity, were also studied: ‘Butler’, ‘Camponica’, ‘Daviana’, ‘Ennis’, ‘Gironell’, ‘Grande’, ‘Kalinkara’, ‘Morell’, ‘Mortarella’, ‘Negret’, ‘Riancho’, ‘Royal’, ‘Segorbe’, ‘Tombul’, and ‘Tonda di Giffoni’. In addition, the accessions ‘Araujo’ and ‘Avellanosa’ collected in Spain were included too. These 17 accessions were considered as non-local accessions. All accessions were obtained by vegetative propagation (rooted sucker) from the original tree.

### Phenotyping

The morpho-agronomic characterization included eight phenological descriptors and nine kernel and nut descriptors (Table 1). The descriptions of these characteristics were based on a list of standardized hazelnut descriptors (Bioversity FAO and CIHEAM, 2008). To record the phenological characteristics, the field collection was visited between December 1 and August 30 over three consecutive years (2017, 2018 and 2019). Phenological data were taken from two trees of each accession (clones). Supplementary Figure 1 shows the climatic condition recorded during the 3 years. To record the morphological traits of kernel and nuts, 70–100 hazelnuts per tree were manually harvested and placed in the shade until characterized.

**TABLE 1:** List and description of the 17 characters considered in the morpho-agronomic characterization of the 58 hazelnut accessions and cultivars.

Traits	Abbreviation	Unit	Description 1
**Phenological**			
First male bloom date	FF_Male	Days	Days until the first catkins (> 5%) are open and produce pollen
Last male bloom date	LF_Male	Days	Days until the last catkins are senesce and do not produce pollen
Male flowering period	PF_Male	Days	LF_Male-FF_Male
First female bloom date	FF_Female	Days	Days until the first female blooms (> 5%) are open and purple stigmata can be observed
Last female bloom date	LF_Female	Days	Days until the last female blooms (< 5%) are open
Female flowering period	PF_Female	Days	LF_Female - FF_Female
Days to vegetative budbreak	Vbudbreak	Days	Days until the vegetative buds are open and first leaves can be observed
Nut maturity date	Harvest	Days	Days until nut shells show color
**Morphological**			
Nut length	NutL	mm	Average of at least 25 nuts, measured from most distant points along main seed axis
Nut width	NutW	mm	Average of at least 25 nuts, measured on the widest point perpendicular to main seed axis
Nut thickness	NutT	mm	Average of at least 25 nuts, measured at the widest part perpendicular to cotyledon suture
Nut weight	NutWe	g	Average of at least 25 nuts
Kernel length	KernelL	mm	Average of at least 25 kernels, measured from most distant points along main seed axis
Kernel width	KernelW	mm	Average of at least 25 kernels, measured on the widest point perpendicular to main seed axis
Kernel thickness	KernelT	mm	Average of at least 25 kernels, measured at the widest part perpendicular to cotyledon suture
Kernel weight	KernelWe	g	Average of at least 25 kernels
Kernel percentage	KernelP	%	(kernel dry weight × 100)/nut dry weight

The broad-sense heritability (*H*^2^) for each trait was estimated using the repeatability function of the ‘heritability’ package in R software (Kruijer et al., 2015). *H*^2^ was estimated at the genotypic level in accordance with the equation *Vg*/(*Vg* + *Ve*/*r*), where *Vg* = [*MS*(*G*)–*MS*(*E*)]/*r*, *Ve* = *MS*(*E*), *r* represents the number of replicates per genotype, *MS*(*G*) represents the mean sum of squares for genotype and *MS*(*E*) represents the mean sum of squares for residual error obtained from the analysis of variance.

### Chemical Analyses

The nut samples were manually cracked and 50 g of kernels were finely milled in a Pulverisette 14 mill (Fritsch, Idar-Oberstein, Germany) with a 1.0-mm sieve. Analyses were performed on 47 hazelnuts cultivars and selections harvested in 2019.

#### Proximate Composition

Moisture was determined by drying at 105°C, ash by incineration at 550°C, crude protein by Kjeldahl method and total fat by Shoxlet method according to AOAC methods (Horwitz, 2005). Total carbohydrate and energy were indirectly calculated in accordance with FAO (2003). Total carbohydrate (%) was estimated using the difference method as follows:

Total carbohydrate (%) = 100 − (% protein + % fat + % water + % ash), and the energy content was calculated as follows:

Energy (kcal/100 g) = 4 × (crude protein g) + 4 × (carbohydrate g) + 9 × (crude fat g).

#### Oil Analysis

Oils were obtained from milled hazelnuts (10 g) by extraction with petroleum ether (b.p. 40–60°C) in a Soxhlet apparatus. The oils were stored at 4°C under nitrogen for further analyses.

Fatty acids were analyzed by gas chromatography (GC) after conversion into their corresponding methyl esters using a cold methanolic solution of potassium hydroxide in accordance with the International Olive Oil Council, (2001). Hundred microgram of sample was dissolved in 3 mL hexane and mixed vigorously for 45 s with a 0.5 mL methanolic solution. After 30 min, the hexane phase was recovered and analyzed by GC in an Agilent 7,980 chromatograph equipped with an MSD 5975C (Palo Alto, CA, United States) and a J&W CP 7420 FAME column (100 m × 250 μm and 0.25-μm i.d). Oven conditions were as follows: 180°C (10 min) rising to 200°C (25 min) at a rate of 1°C/min and rising to 240°C (10 min) at a rate of 15°C/min. Analysis were carried out in split mode (1/100) and the carrier gas flow (He) was set at 1 mL/min. All analyses were carried out in duplicate.

Squalene was analyzed by GC following the method described by Lanzón et al. (1995). Briefly, 200 mg of oil were dissolved in 5 mL hexane, mixed with 1 mL of a squalene solution (1 mg/mL, internal standard) and 1 mL of methanolic solution of potassium hydroxide (2 N) and then shaken vigorously for 1 min. After 10 min, the organic phase was washed twice with ethanol:water (50:50), and 1 μL was analyzed by GC in the same equipment described above with a HP-5MS column (30 m × 250 μm and 0.25-μm i.d.). Oven conditions were as follows: 250°C (15 min) rising to 300°C (10 min) at a rate of 20°C/min. The analyses were carried out in split mode (1/10), and the carrier gas flow was set at 1 mL/min. All the analyses were carried out in duplicate.

Tocopherol isomers were determined by high performance liquid chromatography (HPLC) with fluorescence detection at an excitation wavelength of 298 nm and an emission wavelength of 345 nm following Rodríguez Madrera and Suárez Valles (2018). Aliquots of oil samples (100 mg) were dissolved in 2 mL of isopropanol and filtered through 0.22 μm PVDF membranes (Teknokroma, Barcelona, Spain) prior to injection into the HPLC system. The HPLC analyses were performed on a Waters system (Waters Corporation, Mildford, MA, United States), equipped with a 717 automatic and programmable temperature injector at 12°C, a M510 pump, a column oven (23°C) and a 2,475 fluorescence detector. The separation of tocopherols was carried out on a Fluophase PFP column (250 × 4 mm, 5 μm) from Thermo-Fisher Scientific (Waltham, MA, United States), in isocratic mode using methanol:water (90:10) as the mobile phase at a flow rate of 1 mL/min and an injection volume of 10 μL. Quantitation was performed using the external standard method. All the analyses were carried out in duplicate.

### Statistical Analyses

Statistical analyses were carried out using R software (R Core Team, 2020). Mean values were adjusted by identifying outliers using the coefficient of variation [CV = (standard deviation/mean)^∗^100]. A coefficient of variation higher than 25% was not accepted, and the outliers were removed prior to the statistical analysis. Pearson’s correlation coefficients among the traits were determined using the package corrplot (Wei and Simko, 2017). Student’s *t*-tests were used for the identification of significant differences between the local and non-local accessions. A hierarchical clustering on principal components (HPCP) analysis was conducted to identify the main clusters and to visualize the data structure. The HPCP was conducted in R software using the packages ggplot2 (Wickham, 2016), FactoMineR and FactoExtra (Lê et al., 2008). Significant differences between the identified clusters were investigated by Tukey tests for each character evaluated.

## Results

### Variation for Phenological Traits

**FIGURE 1:**
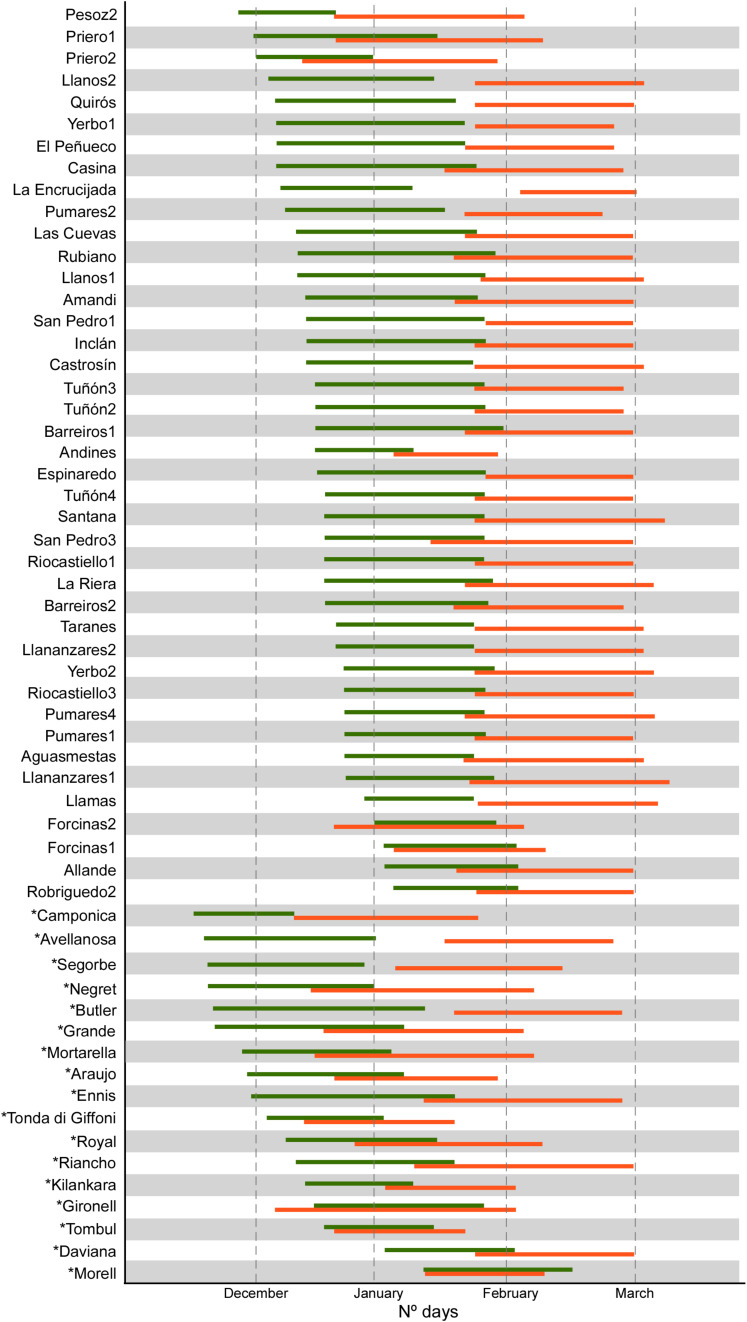
Period of male (green) and female flowering (orange) of 58 hazelnut accessions at SERIDA in Villaviciosa, Spain. December 1 was taken as day one in the evaluation in each year.

The results showed wide ranges for the eight phenological traits evaluated in 58 accessions (Figure 1 and Supplementary Table 1). For instance, FF_Male, FF_Female and Vbudbreak ranged from 16 (observed in ‘Camponica’) to 70 days (‘Morell’), from 40 (‘Camponica’) to 85 days (‘Daviana’, ‘Espinaredo’, and ‘San Pedro 1’) and from 78 (‘Camponica’) to 120 days (‘Robriguedo’), respectively. Significant correlations were detected between most phenological characteristics, except for those involved Harvest date, and PF_Female with PF_Male, LF_Female and LF_Female (Supplementary Figure 2A). Estimations of *H*^2^ (Table 2) were high for most traits (>0.75) but moderate for FF_Male (0.65), PF_Male (0.40), PF_Female (0.31), and KernelP (0.33).

**TABLE 2:** Means and standard errors (SE) for phenological and morphological traits evaluated in local and non-local hazelnut accessions at SERIDA in Villaviciosa, Spain.

	¡	Local			Non-local				
			
Traits	Abbreviation	Mean		SE	Mean		SE	T-St	*H*^2^
**Phenological**									
First male bloom date (days)	FF_Male	45.1	±	1.4	34.5	±	3.9	s	0.65
Last male bloom date (days)	LF_Male	82.1	±	1.4	69.9	±	3.7	s	0.79
Male flowering period (days)	PF_Male	37.1	±	1.0	35.4	±	2.0	ns	0.40
First female bloom date (days)	FF_Female	76.5	±	1.8	56.9	±	3.6	s	0.92
Last female bloom date (days)	LF_Female	116.1	±	1.7	99.3	±	3.3	s	0.95
Female flowering period (days)	PF_Femele	39.6	±	0.8	42.4	±	1.9	ns	0.31
Days to budbreak (days)	Vbudbreak	111.9	±	1.3	97.7	±	3.0	s	0.92
Nut maturity date (days)	Harvest	249.4	±	0.4	248.0	±	0.9	ns	-
**Morphological**									
Nut length (mm)	NutL	1819.7	±	12.5	2157.6	±	65.8	s	0.95
Nut width (mm)	NutW	1729.5	±	8.8	2036.0	±	62.7	s	0.97
Nut thickness (mm)	NutT	1549.8	±	12.7	1745.7	±	58.8	s	0.98
Nut weight (g)	NutWe	18.5	±	0.3	25.0	±	1.9	s	0.76
Kernel length (mm)	Kernel	1396.1	±	13.3	1600.9	±	58.5	s	0.98
Kernel width (mm)	KernelW	1279.7	±	48.1	1408.2	±	159.0	s	0.93
Kernel thickness (mm)	KernelT	1173.9	±	12.3	1249.1	±	37.6	s	0.96
Kernel weight (g)	KernelWe	9.4	±	0.1	10.8	±	0.7	s	0.69
Kernel percentage (%)	Kernel%	50.7	±	0.6	43.9	±	1.1	s	0.33

*Estimates of broad-sense heritability (H^2^) for each trait are also included. T-St, T-student test; s, significant difference (p < 0.05); ns, non-significant difference (p > 0.05).*

**FIGURE 2:**
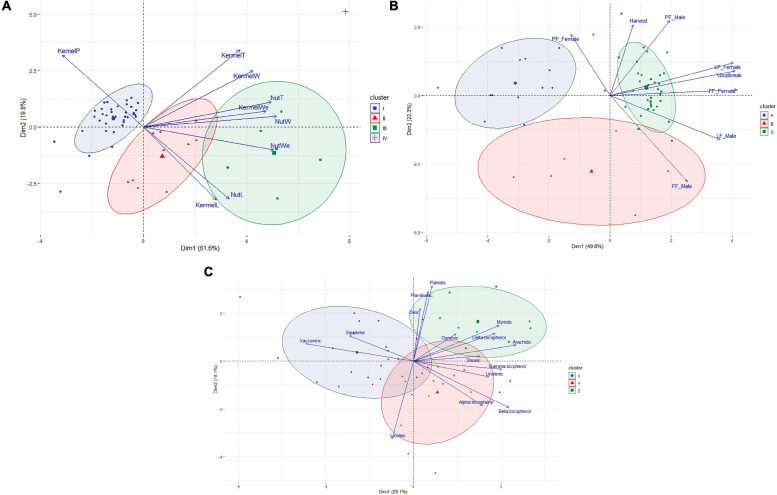
Biplots showing the results of the Hierarchical Clustering on Principal Components analysis for the three sets of evaluated traits. **(A)** Biplot showing the distribution of the 56 hazelnut accessions considering the eight phenological traits and obtained from two main estimated dimensions. **(B)** Biplot showing the distribution of the 56 hazelnut accessions considering the nine morphological characters of hazelnut and obtained from two main estimated dimensions. **(C)** Biplot showing the distribution of the 47 hazelnut accessions considering the chemical composition of their oil and obtained from two main estimated dimensions. Ellipses representing the clusters were drawn considering, *p* > 0.8.

The HPCP analysis using the averages of the eight phenological traits revealed three main clusters and two main dimensions explaining 72.1% of the variance (Figure 2A and Supplementary Table 1). Cluster A included 13 accessions, ten of which were reference cultivars, that had significantly lower values for FF_Male, LF_Male, FF_Female, and Vbudbreak. Cluster B contained six accessions with intermediate values for FF_Male and FF_Female. Cluster C included 37 accessions, of which most (33) were local selections, that had significantly higher values for FF_Male, LF_Male, PF_Male, FF_Female, and Vbudbreak.

### Variation for Morphological Characters of Hazelnut

There were wide variations in the nine evaluated morphological characteristics in 56 accessions (data non-available in 2 accessions; Supplementary Table 2). For instance, NutWe, KernelWe, and KernelP ranged from 14.2 (observed in ‘Daviana’) to 37.9 g (‘Ennis’), from 4.1 (‘Daviana’) to 14.9 g (‘Ennis’) and from 38.7 (‘Royal’) to 54.9 g (‘Llananzares’), respectively. Significant correlations were found among most of the evaluated traits, except for KernelT with three traits (KernelP, NutL, and KernelL) and, KernelW with NutL and KernelL (see Supplementary Figure 2B). Significant differences were observed between local and non-local materials for the nine morphological traits (Table 2). Estimations of *H*^2^ were high (>0.65) for most traits except for KernelP (0.33).

The HPCP analysis revealed three main clusters (without considering the cultivar ‘Kalinkara’ in Cluster IV) as well as two main dimensions that explained 81.4% of the variance (Figure 2B and Supplementary Table 2). Cluster I included 39 accessions, most being local accessions (37), having significantly lower values for NutL, NutW, KernelL, and NutWe but with a significantly higher value for KernelP. These accessions all produce small hazelnuts. Cluster II contained 10 accessions, most being reference accessions (9), having intermediate values and being significantly different from the other two groups for NutL, NutW, KernelL, and NutWe. Cluster III included six reference accessions having significantly higher values for most traits, except for KernelP.

### Variation in Chemical Traits

The proximate composition (fat, protein, carbohydrate, moisture and ash) of 39 local accessions and eight reference cultivars were analyzed (Supplementary Table 3). Fat was the main constituent of the kernels (mean 64%), followed by carbohydrate (17%) and protein (12%). Total fat in local varieties ranged between 57.7% (‘Allande 3’) and 68.8% (‘Priero 2’), with a mean value of 64.5%. In total, 10 fatty acids (Supplementary Table 4)—myristic acid (C14:0), palmitic acid (C16:0), palmitoleic acid (C16:1n-7), stearic acid (C18:0), oleic acid (C18:1n-9), vaccenic acid (C18:1n-7), linoleic acid (C18:2n-6), linolenic acid (C18:3n-3), arachidic acid (C20:0), and gondoic acid (C20:1n-9)—were detected in all the oils studied. Oleic acid was the major unsaturated fatty acid in all the samples (mean 76.97%), followed by linoleic acid (mean 11.34%) and vaccenic acid (mean 1.50%), and the major saturated fatty acids were palmitic acid (mean 7.23%) and stearic acid (mean 2.64%). Other fatty acids were present at less than 1%. The greatest variability in the fatty acid content was observed for the major compounds, highlighting the range of variability between local varieties for oleic acid, between 73.5% (‘Espinaredo’) and 79.3% (‘Robriguedo 2’), and for linoleic acid, between 8.2% (‘Tuñon 2’) and 15.6% (‘Espinaredo’).

The highest total tocopherol content was detected in ‘Espinaredo’ (629.5 mg/kg) and the lowest in ‘Priero 2’ (332.8 mg/kg) (Supplementary Table 5). α-Tocopherol was the most abundant isomer (>89.5% total tocopherols), with concentrations ranging between 319.4 mg/kg (‘Priero 2’) and 586.2 mg/kg (‘Barreiros 2’). Finally, the content of squalene, another component of hazelnut with antioxidative activities, was also analyzed. The local varieties showed an average content of 243 mg/kg, and a wide range from 612.3 mg/kg (‘Casina’) to 199.0 mg/kg (‘Santana 2’).

No statistical differences in the analyzed chemical compounds were detected between the local and non-local accessions, with the exception of the ash, crude protein, carbohydrate and stearic acid content (Table 3). An HPCP of the chemical composition of oils revealed three main clusters and two main dimensions that explained 45.2% of the variance (Figure 2C and Supplementary Table 3): Cluster X included 19 accessions having significantly lower values for linolenic acid, arachidic acid and γ-tocopherol but with significantly higher vaccenic acid values. Cluster Y contained 18 accessions, which did not differ significantly from the other two clusters in the composition of any parameter analyzed. Cluster Z included 10 accessions having significantly higher values for myristic acid, palmitoleic acid, gondoic acid and δ-tocopherol.

**TABLE 3:** Means and standard errors (SE) for chemical characterization of local and non-local accessions at SERIDA in Villaviciosa, Spain.

	Local			Non-local			
			
Chemical composition	Mean		SE	Mean		SE	Student’s *t-*tests
**Proximate composition**							
Moisture (%)	4.22	±	0.05	4.18	±	0.16	ns
Ash (%)	2.14	±	0.02	2.35	±	0.09	s
Crude protein (%)	11.80	±	0.19	13.03	±	0.63	s
Fat (%)	64.54	±	0.39	64.89	±	0.59	ns
Carbohydrates (%)	17.30	±	0.29	15.54	±	0.75	s
Energy (Kcal)	697.26	±	2.15	698.35	±	3.40	ns
**Oil composition**							
Myristic acid (%)	0.02	±	0.00	0.02	±	0.00	ns
Palmitic acid (%)	7.23	±	0.09	7.03	±	0.11	ns
Plamitoleic acid (%)	0.15	±	0.00	0.15	±	0.01	ns
Stearic acid (%)	2.64	±	0.06	2.32	±	0.08	s
Oleic acid (%)	76.97	±	0.25	78.16	±	0.76	ns
Vacceninc acid (%)	1.50	±	0.02	1.54	±	0.03	ns
Linoleic acid (%)	11.34	±	0.27	10.63	±	0.72	ns
Linolenic acid (%)	0.04	±	0.00	0.04	±	0.00	ns
Arachidic acid (%)	0.06	±	0.00	0.06	±	0.00	ns
Gondoic acid (%)	0.05	±	0.00	0.06	±	0.00	ns
Aqualene (mg/kg)	294.36	±	16.38	301.92	±	20.68	ns
α-tocopherol (mg/kg)	473.34	±	11.89	475.31	±	32.45	ns
β-tocopherol (mg/kg)	13.53	±	0.48	11.08	±	1.13	ns
γ-tocpherol (mg/kg)	24.23	±	1.06	19.84	±	2.72	ns
δ-tocopherol (mg/kg)	2.87	±	0.24	2.35	±	0.27	ns

*s, significant difference (p < 0.05); ns, non-significant difference (p > 0.05).*

## Discussion

Morpho-agronomic evaluations under field conditions are necessary steps for the identification and differentiation of genotypes with superior features in germplasm collections or breeding progenies owing to environmental effects on expression of traits. In this work, the phenology, nut morphological traits and kernel chemical composition of 39 accessions collected in Asturias and 17 non-local accessions were evaluated. Estimates of *H*^2^ were high for the phenological and nut morphological traits evaluated, indicating the high genetic bases of their variability. These high *H*^2^ estimates agreed with those previously reported (Yao and Mehlenbacher, 2008).

Observed phenological traits revealed that male and female flowering occurred at different times (dichogamy) with a limited overlap, except for the local accessions ‘Forcinas 1’ and ‘Forcinas 2’ and the reference cultivar ‘Morell’ (Figure 1). The phenological behaviors of the reference cultivars ‘Butler’, ‘Negret’, ‘Ennis’, ‘Tombul’, and ‘Morell’ agreed with those previously reported (Bioversity FAO and CIHEAM, 2008). Most accessions exhibited a male flowering period before the female flowering period (protandry). In addition, the local accessions had very similar phenological traits and exhibited late flowering (Figure 1) compared with the well-known cultivars ‘Camponica’, ‘Negret’ and ‘Tonda di Giffoni’. Reference cultivars did not show a large overlap with the local accessions between the male and female flowering periods, except in the case of ‘Morell’. This finding must be considered when designing new plantations of local accessions, particularly in northern Spain, owing to hazelnut’s particular pollination system.

The evaluation of nut traits grouped most of the local accessions together (Figure 2B). They had smaller nuts and kernels than the reference cultivars but with higher kernel percentages. Local accessions also produced round-shaped nuts having similar values for NutL, NutW and NutT (see Supplementary Table 2). Round-shaped nuts and high kernel percentage are main objectives of hazelnut breeding (Botta et al., 2019); therefore, these local materials are an interesting source of these traits.

The analysis of the kernel chemical composition revealed values for ash, proteins, fat and carbohydrates that were within previously reported ranges. The observed protein values agree with those described for distinct varieties of hazelnut cultivated in Portugal (Amaral et al., 2006; mean 10.9%) and Poland (Krol et al., 2019; 12.4%) and lower than reported for varieties cultivated in Turkey (Köksal et al., 2006; 17.4%) and Iran (Hosseinpour et al., 2013; 19.5%). Likewise, a significant difference (Table 3) was detected between the local accessions and the reference varieties, which indicated that, regardless of the growing conditions, the local varieties produced fruits with lower protein contents.

The reported lipid content values revealed a large degree of variability among cultivars that was attributed to genotypic and geographical factors (Savage and McNeil, 1998; Bacchetta et al., 2013). In general, local accessions were satisfactory oil producers, whether compared with the reference cultivars (mean value: 64.9% ± 1.7%) or with the results for other cultivars (Amaral et al., 2006; Oliveira et al., 2008). We investigated possible differences in the fat content owing to geography. The local cultivar ‘Casina’ and the reference cultivars ‘Butler’, ‘Segorbe’, ‘Morell’, and ‘Negret’ showed similar or higher oil content than reported in Argentina (Cittadini et al., 2020), New Zealand (Savage and McNeil, 1998), Iran (Hosseinpour et al., 2013), Italy (Bacchetta et al., 2013), and Portugal (Amaral et al., 2006).

Tocopherols are constituents of vitamin E, a potent antioxidant that plays an important role in preventing age-related diseases, cardiovascular diseases, cancer, diabetes and obesity (Shahidi and de Camargo, 2016). The observed tocopherol values were in accordance with data reported for other cultivars distributed worldwide (Savage and McNeil, 1998; Parcerisa et al., 1998; Köksal et al., 2006; Bacchetta et al., 2013; Fernandes et al., 2017; Cittadini et al., 2020). No statistical differences were detected between the local accessions (total tocopherol mean: 514.0 ± 81.2 mg/kg) and the references (mean total tocopherols: 508.6 ± 93.1 mg/kg).

Squalene is another component of hazelnut with antioxidative activities and other bioactive properties (Lozano-Grande et al., 2018). Although there is limited available information on squalene in hazelnut oil, the levels detected here were in accordance with data reported by various authors (Benitez-Sánchez et al., 2003; Fernandes et al., 2017; Cittadini et al., 2020), and higher than that reported by Maguire et al. (2004), although the influences of many factors, such as the place of origin, crop management and storage conditions, must be taken into account.

Finally, considering the groups established in the HPCA analysis from nut and phenological traits, eight local accessions can be highlighted (Supplementary Figure 3); ‘Pesoz-2’, ‘Priero-1’, ‘Priero-2’, ‘Andines-2’, ‘Forcinas-1’, and ‘Forcinas-2’ which significantly differ from the rest of local accessions for phenological data and significant precocity for flowering female (FF_Female); ‘Allande-3’ and ‘Pumares-2’ which significantly differ from the remining local accessions for morphological nut data due to intermediate values for NutL, KernelL, and NutWe. The accessions ‘Forcinas-1’, and ‘Forcinas-2’ showed a high overlap between the male and female flowering periods (see Figure 1). Thus, these eight genotypes are a high priority for preservation and further evaluation for planting on a larger scale. The remaining local accessions did not exhibit significant differences in nut and phenological traits and were, therefore, placed in a group, tentatively named the ‘Casina group’. This ‘Casina group’ is characterized by the production of small round hazelnuts, with high proportions of kernel, and late male and female flowering.

## Summary

Morphological and phenological evaluations of 41 local accessions collected in Asturias (northern Spain) and 17 non-local accessions maintained in the SERIDA collection revealed that the local accessions produced smaller nuts and kernels than the reference cultivars, but with higher kernel percentage. The local accessions generally exhibited significantly later male and female flowering compared with the reference cultivars. However, non-significant differences were detected between the two groups for most chemical components of nuts. Within the local materials it was possible to differentiate eight accessions from the remaining local accessions, including the ‘Casina’ landrace. These local cultivars and selections will be useful for breeding new cultivars.

## Data Availability Statement

The original contributions presented in the study are included in the article/Supplementary Material, further inquiries can be directed to the corresponding author/s.

## Author Contributions

AC performed the phenotyping and statistical data analysis. RR and BS performed the chemical analyses. JJF conceived and prepared the manuscript and conducted statistical data analysis. All authors read and approved the last version.

## Conflict of Interest

The authors declare that the research was conducted in the absence of any commercial or financial relationships that could be construed as a potential conflict of interest.

## Supplementary Material

Supplementary Figure 1 Temperature and rainfall recorded during 2017, 2018 and 2019 in a weather station locates close to the field in which is located the hazelnut collection (43°29′33″N–05°14′50″W).

Supplementary Figure 2 Corrplots showing the observed correlations for the three set of evaluated traits. **(A)** Corrplot for phenological traits. **(B)** Corrplot for hazelnut fruit traits Non-significant correlations are indicated using a cross.

Supplementary Figure 3 Hazelnut phenotype of the eight accessions differentiated from ‘Casina group’ in this study. **(A)** ‘Allande-3’. **(B)** ‘Andines-2’. **(C)** ‘Casina’. **(D)** ‘Forcinas-1’. **(E)** ‘Forcinas-2’. **(F)** ‘Pesoz-2’. **(G)** ‘Priero-1’. **(H)** ‘Priero-2’. **(I)** ‘Pumares-2’.

Supplementary Table 1 Means of phenological traits recorded during 3 years. Cluster in which each accession was included from the HCPC analysis is indicated. ^∗^Non-local accessions.

Supplementary Table 2 Mean of nut morphological traits recorded during 3 years. Cluster in which each accession was included from the HCPC analysis is indicated. ^∗^Non-local accessions.

Supplementary Table 3 Proximate composition of local and reference varieties. Cluster in which each accession was included from the HCPC analysis is indicated. ^∗^Non-local accessions.

Supplementary Table 4 Fatty acid composition (%) of oils extracted from local and reference cultivars. ^∗^Non-local accessions.

Supplementary Table 5 Squalene and tocopherol content in oils extracted from local and reference varieties (mg/kg). ^∗^Non-local accessions.
