# Supplementary material for: Variation of Morphological, Agronomic and Chemical Composition Traits of Local Hazelnuts Collected in Northern Spain
Source: Front Plant Sci. 2021 Jun 15;12:659510. doi: 10.3389/fpls.2021.659510 (PMC8239416; doi:10.3389/fpls.2021.659510)
Supplement: Supplementary Figure 1 — Temperature and rainfall recorded during 2017, 2018 and 2019 in a weather station locates close to the field in which is located the hazelnut collection (43°29′33″N–05°14′50″W). [file Data_Sheet_1.PDF]

SUPPLEMENTARY MATERIAL

Variation of morphological, agronomic and chemical composition traits of local hazelnuts collected in northern Spain

Ana Campa Negrillo, Plant Genetic Group, Regional Service for Agrofood Research and Development (SERIDA), 33300, Villaviciosa, Asturias, Spain. acampa@serida.org
https://orcid.org/0000-0003-3970-9079

Roberto Fernández-Madrera, Food Technology Area, Regional Service for Agrofood Research and Development (SERIDA), 33300, Villaviciosa, Asturias, Spain. rrodriguez@serida.org

Belén Suárez Valles, Food Technology Area, Regional Service for Agrofood Research and Development (SERIDA), 33300, Villaviciosa, Asturias, Spain. mbsuarez@serida.org

Juan Jose Ferreira, Plant Genetic Group, Regional Service for Agrofood Research and Development (SERIDA), 33300, Villaviciosa, Asturias, Spain. jjferreira@serida.org
https://orcid.org/0000-0002-8782-8868

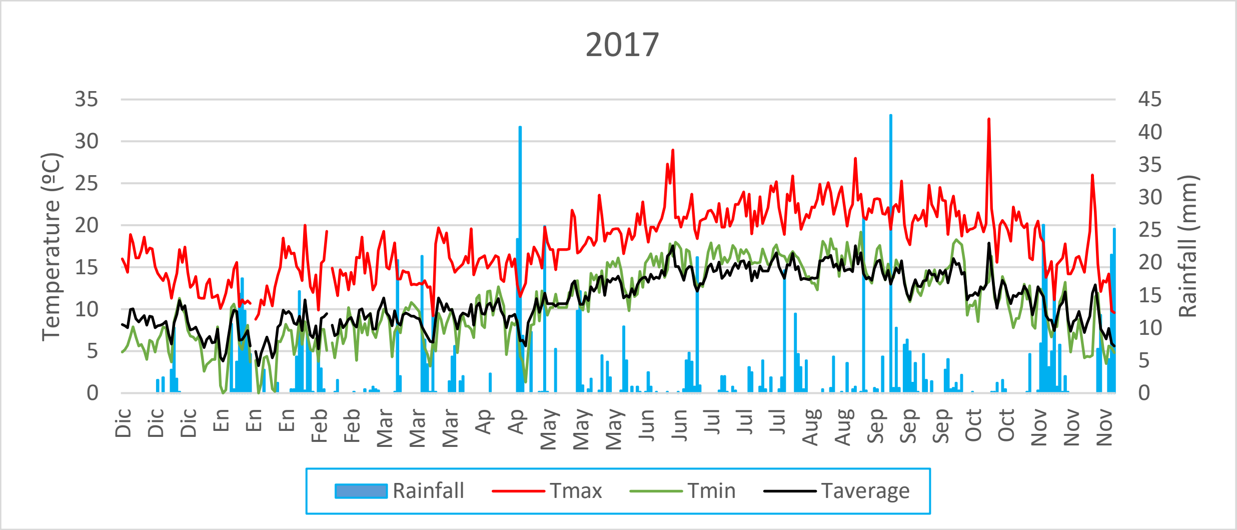

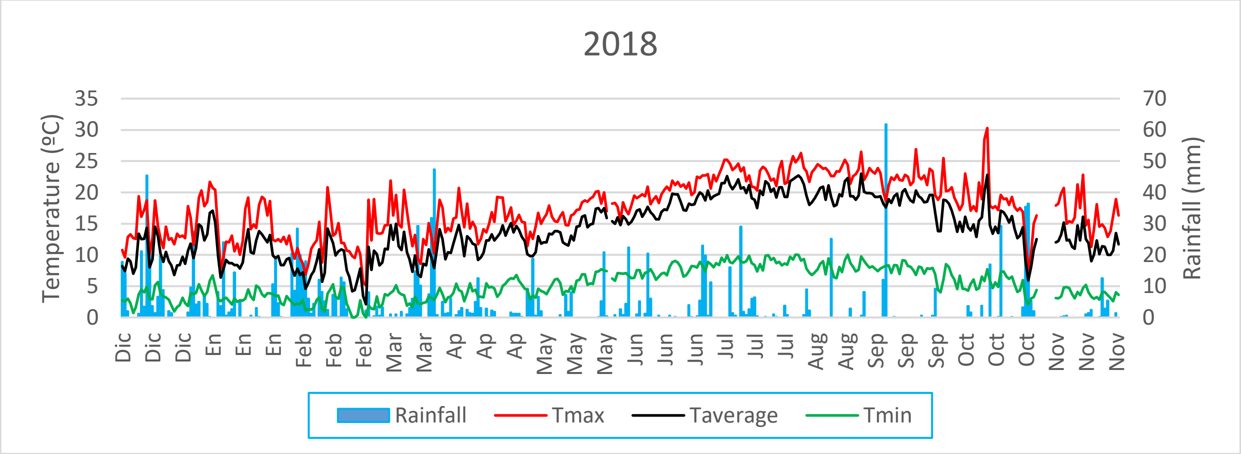

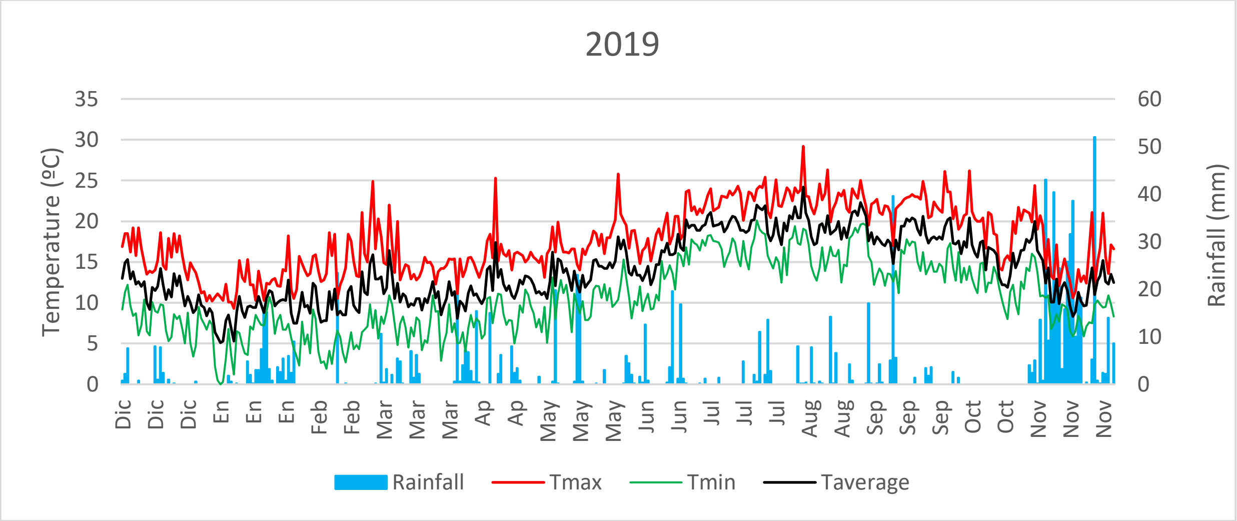

Supplementary Figure 1 Temperature and rainfall recorded during 2017, 2018 and 2019 in a weather station locates close to the field in which was the hazelnut collection (43º29'33''N - 05º14'50''W).

A

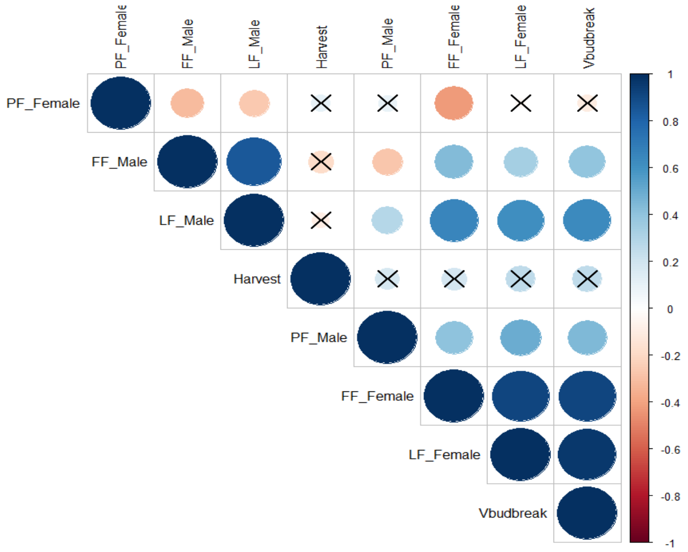

B

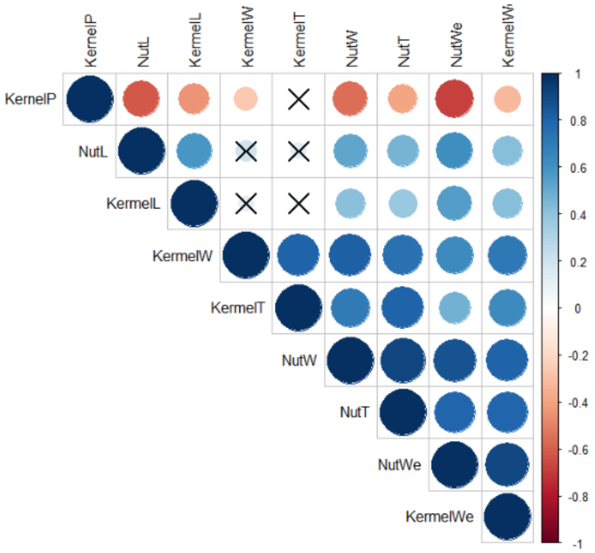

Supplementary Figure 2 Corrplots showing the observed correlations for the three set of evaluated traits. A/ Corrplot for phenological traits. B /Corrplot for hazelnut fruit traits Non significant correlations are indicated using a cross.

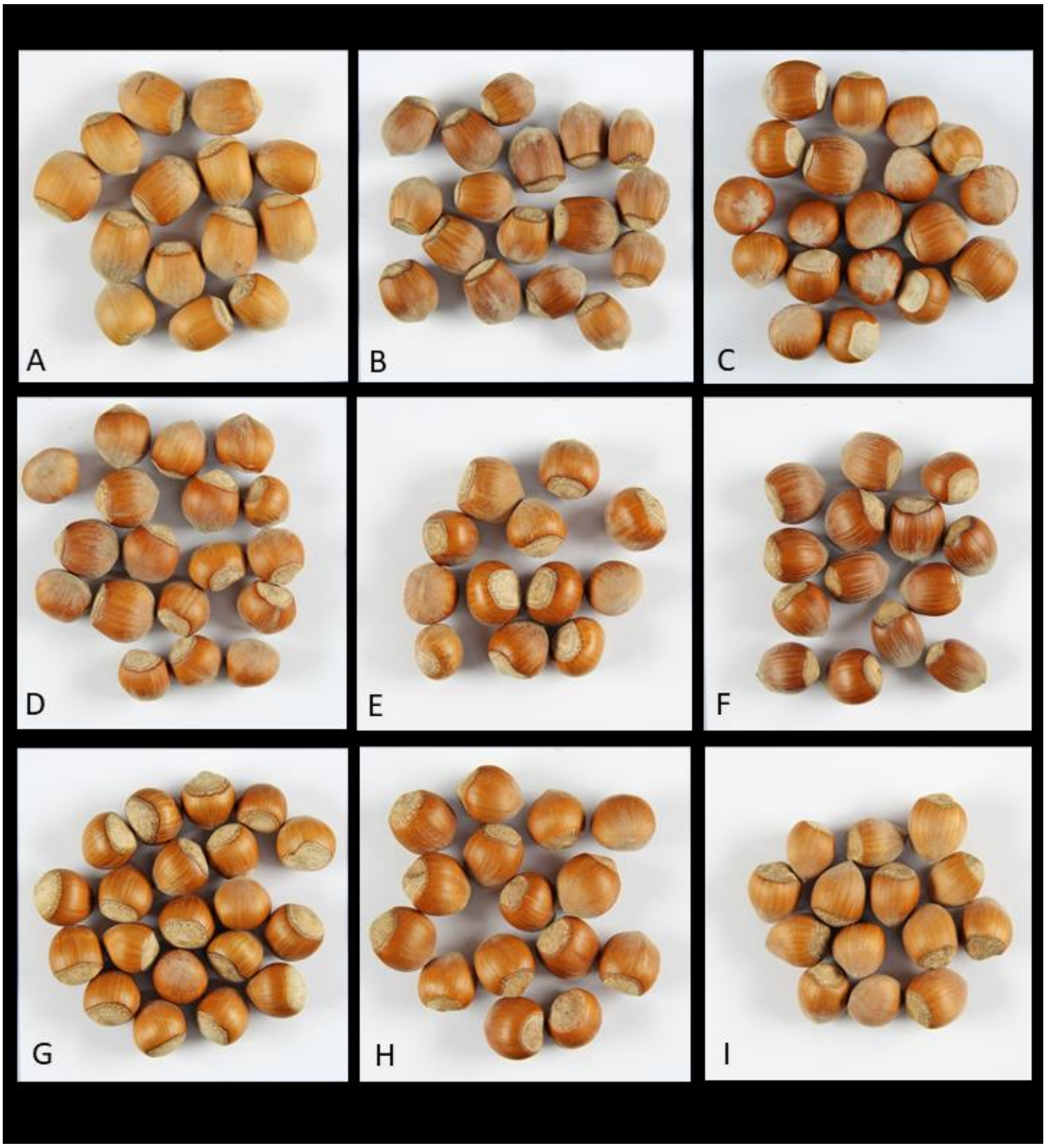

Supplementary Figure 3. Hazelnut phenotype of the eight accessions differentiated from 'Casina group' in this study. A/ 'Allande-3'. B/ 'Andines-2'. C/ 'Casina'. D/ 'Forcinas-1'. E/ 'Forcinas-2'. F/ 'Pesoz-2'. G/ 'Priero-1'. H/ 'Priero-2'. I/ 'Pumares-2'.

Supplementary Table 1 Means of phenological traits recorded during three years. Cluster in which each accession was included from the HCPC analysis is indicated. *, non-local accessions

Accession	FF_Male	LF_Male	PF_Male	FF_Female	LF_Female	PF_Female	Vbudbreak	Harvest	Cluster
Aguasmestas-1	52	82	30	80	122	42	115	244	C
Allande-3	61	96	35	78	120	42	117	249	C
Amandi	42	83	40	78	120	42	112	249	C
Andines-2	45	68	23	63	88	24	96	245	B
Araujo*	29	66	37	49	88	38	88	248	A
Avellanosa*	19	59	40	75	115	40	112	252	C
Barreiros-1	45	89	44	80	120	40	115	249	C
Barreiros-2	47	85	38	77	117	40	115	245	C
Butler*	21	71	50	77	117	40	110	255	C
Camponica*	16	40	24	40	83	43	78	248	A
Casina	36	83	47	75	117	42	112	249	C
Castrosin-2	43	82	39	82	122	40	112	249	C
Daviana*	64	93	29	85	117	33	117	245	B
El Peñueco-1	36	80	44	80	115	35	110	246	C
Enis*	30	78	48	70	117	47	115	245	C
Espinaredo	45	85	40	85	120	35	112	247	C
Forcinas-1	61	92	31	63	99	36	103	255	B
Forcinas-2	59	87	29	49	94	45	96	245	B
Gironell*	45	85	40	40	92	52	90	250	A
Grande*	21	66	44	47	94	47	94	247	A
Inclan	43	85	42	82	120	37	110	252	C
Kilankara*	47	66	19	47	94	47	96	250	A
LaRiera-2	47	87	40	80	124	44	117	251	C
LasCuevas-1	44	85	41	81	122	41	116	250	C
Llananzares-1	52	87	35	80	127	47	120	249	C
Llananzares-2	50	82	32	82	122	40	117	250	C
Llanos-1	41	85	44	82	122	40	120	255	C
Llanos-2	34	73	39	82	122	40	120	254	C
Morell*	70	105	35	71	99	28	98	240	B
Mortarella*	28	63	35	45	96	52	96	250	A
Negret*	20	59	39	44	96	53	90	248	A
Pesoz-2	27	50	23	49	94	45	92	248	A
Priero-1	30	73	43	50	98	49	98	247	A
Priero-2	31	58	27	42	88	46	88	249	A
Pumares-1	52	85	33	82	120	37	117	251	C
Pumares-2	38	75	38	80	112	32	112	247	C
Pumares-4	52	85	33	80	125	45	120	252	C
Quiros	35	78	42	82	120	37	115	255	C
Riocastiello-1	47	85	38	82	120	37	112	249	C
Riocastiello-3	52	85	33	82	120	37	110	251	C
Roancho*	40	78	37	68	120	52	110	243	C
Robriguedo-2	63	92	29	83	120	37	120	249	C
Royal*	36	71	35	54	98	44	98	249	A
Rubiano-1	41	87	46	77	120	42	115	245	C

SanPedro-1	43	85	42	85	120	35	117	247	C
SanPedro-3	47	85	38	72	120	48	115	252	C
Santana-2	47	85	38	82	127	45	120	252	C
Segorbe*	20	56	37	64	103	39	108	252	A
Taranes-2	50	82	32	82	122	40	117	249	C
Tombul*	47	73	26	49	80	31	80	245	B
TondaGiffoni*	33	61	28	42	78	35	80	250	A
TuÏ½n-2	45	85	40	82	117	35	112	250	C
Tuñon-3	45	85	40	82	117	35	110	249	C
Tuñon-4	47	85	38	82	120	37	112	249	C
Yerbo-1	36	80	44	82	115	33	110	251	C
Yerbo-2	52	87	35	82	124	42	115	251	C

Supplementary Table 2 Mean of nut morphological traits recorded during three years. Cluster in which each accession was included from the HCPC analysis is indicated.*, non-local accessions.

**Accession**	**NutL**	**NutW**	**NutT**	**KernelL**	**KernelW**	**KernelT**	**NutWe**	**KernelWe**	**KernelP**	**Cluster**
Aguasmestas-1	1779.2	1680.5	1560.2	1347.5	1275	1188	16.7	8.6	51.7	I
Allande-3	2057	1683.6	1532.4	1657.2	1214.8	1053.1	22.8	9.7	42.5	II
Amandi	1818.9	1880	1607.5	1352.4	1318	1239.3	19.7	10.4	52.6	I
Andines-2	1913.1	1608.5	1235.7	1503.6	1147.6	885.9	16.8	7.3	43.5	I
Araujo*	2037.3	1979.5	1631.3	1527.1	1409.5	1240.1	27.4	11.1	40.5	II
Avellanosa*	1979.8	1934.7	1644.1	1456.1	1400.5	1202.1	20.9	9	43.3	II
Barreiros-1	1843.2	1760.7	1588.5	1400.8	1270	1146.2	19.6	9.9	50.4	I
Barreiros-2	1771.6	1735.9	1569.5	1344.6	1224.3	1195.4	15	7.7	51.4	I
Butler*	2345.7	2001.2	1743.2	1859.4	1403	1162.3	29.9	13.2	44.2	III
Camponica*	2176.7	2224.9	1783.9	1612.8	1623.2	1235.9	32.3	13.1	40.5	III
Casina	1827.3	1741.3	1598.9	1394.9	1236.2	1193.6	17.9	9.3	52.1	I
Castrosin-2	1738.9	1707.9	1570.4	1344.2	1276.1	1204.7	18.1	9.4	52.1	I
Daviana*	2434.3	1885.4	1684.3	1998.3	1226.7	1125	14.2	6.6	48.3	II
ElPe¤ueco-1	1729.6	1704.7	1554.7	1278.3	1290.5	1213.8	17.7	9.3	52.8	I
Enis*	2524.9	2372	2049.8	1932.1	1495.1	1316.9	37.9	14.9	39.2	III
Espinaredo	1828.5	1708.1	1565.2	1381.9	1286.2	1183.9	18	9.3	51.9	I
Forcinas-1	1735.4	1714	1403.9	1256.2	1245.1	1027.5	18.5	7.2	39.1	I
Forcinas-2	1757.5	1734	1563.7	1346.5	1261.9	1209.4	16.7	8.4	49.9	I
Gironell*	1798.2	1809.4	1558.2	1311.6	1282	1163.6	21.5	8.6	39.8	II
Grande*	2125.8	2380	1918	1614.3	1490.7	1453.7	33	13.6	41.4	II
Inclan	1867.7	1748.8	1602.3	1448.6	1321.1	1221.9	19.9	10.3	51.7	I
Kilankara*	2074.7	2551.4	2262.8	1385.2	1886.8	1754.4	29.9	14.7	49.3	IV
LaRiera-2	1807.5	1738	1589.3	1365	1314.7	1230.7	19.4	10.3	53.3	I
LasCuevas-1	1753.3	1711.8	1593.7	1376.1	1342.3	1273.6	19.7	10.4	52.9	I
Llananzares-1	1742.6	1678.9	1509.8	1352	1294.3	1202	16.8	8.9	52.7	I
Llananzares-2	1758.6	1678.9	1509	1343.7	1243.9	1175.3	16.7	9.2	54.9	I
Llanos-1	1813.6	1747.3	1597.1	1403.1	1310.8	1238.6	19.7	10.4	53	I
Llanos-2	1777.1	1609.7	1323.9	1379	1176	1005.9	14.5	7.8	53.8	I
Morell*	1766.8	1731.8	1523.6	1387.8	1328.6	1108.3	18.7	8.7	46.3	I
Mortarella*	2181.6	1816.7	1494.8	1655.2	1337.8	1141.9	24.5	12.2	49.8	II
Negret*	2029.1	1874.4	1555.8	1538.5	1315.2	1197.5	21.9	10.9	49.5	II
Pesoz-2	1894.8	1685.9	1459.1	1470.1	1280.3	1113.5	18.2	9.7	53.1	I
Priero-1	1745.7	1672.5	1572.8	1334.4	1235	1193.6	16.6	8.1	48.8	I
Priero-2	1894.1	1666.5	1467.9	1461.2	1243.3	1119.3	20	9.3	46.6	I
Pumares-1	1839.9	1740.6	1563.4	1444.9	1267.3	1181.3	19.7	10.2	51.9	I
Pumares-2	2056.3	1749.8	1522.2	1631.4	1179.3	1057.8	21.7	9.1	41.7	II
Pumares-4	1872.2	1722.8	1579.2	1444.6	1312.9	1257	19.7	10.2	51.6	I
Quiros	1791.6	1683	1502.1	1413.4	1297.1	1138	18	9.2	51.3	I
Riocastiello-1	1824.1	1842.4	1610.2	1400.4	1322.9	1207.1	18.8	10.1	53.6	I
Riocastiello-3	1832.1	1789.5	1592	1389	1300.8	1184.4	16.4	8.6	53.3	I
Roancho*	2578.9	2332.5	2074.6	1393.9	1335.9	1227.3	35.9	14.2	39.7	III
Robriguedo-2	1680	1854.6	1630.5	1232.3	1381.9	1222.3	19.6	8.4	42.4	I
Royal*	2690.6	2076.9	1978.6	2134.7	1277	1311.3	34.3	13	38.7	III

Rubiano-1	1716.6	1725.3	1564	1309.2	1279.7	1241.2	16.2	8.5	52.1	I
SanPedro-1	1768.9	1737.8	1605	1345.4	1336	1227.5	19.1	10.2	53.2	I
SanPedro-3	1825.1	1735.7	1599.6	1383	1297.7	1183.2	19.2	9.5	49.5	I
Santana-2	1884	1777.5	1604.5	1446.7	1335.1	1184.7	20	10.4	52.1	I
Segorbe*	2096.4	1920.4	1617.2	1526.6	1439.1	1182.9	26.4	11	41.5	II
Taranes-2	1794.9	1729	1601.1	1330	1300.6	1202.8	18.3	9.7	52.8	I
Tombul*	1930	1619.9	1352.1	1472.1	1258.7	1161.8	14.9	8	53.6	I
Tonda Giffoni*	1908.5	2100.6	1804.1	1409.8	1429.1	1250.3	24.6	10	41.2	II
Tu¤¢n-2	1846.7	1764.4	1594.4	1409.5	1271.2	1164	17.8	9.6	53.7	I
Tu¤¢n-3	1838	1731.1	1577	1384.5	1323.3	1193.6	19.5	10.1	52.1	I
Tu¤¢n-4	1844.7	1746.8	1577.6	1447.6	1291	1197.6	19.2	10.1	52.8	I
Yerbo-1	1869.1	1779.2	1589.4	1445.2	1298.9	1228.6	19.7	10.3	52.3	I
Yerbo-2	1827.1	1743	1555.7	1448	1306.7	1197.7	19.8	10.3	52	I

Supplementary Table 3 Proximate composition of local and reference varieties. Cluster in which each accession was included from the HCPC analysis is indicated. *, non-local accessions

**Accessions**	**Moisture (%)**	**Ash (%)**	**Crude protein (%)**	**Fat (%)**	**Carbohidrates (%)**	**Energy (Kcal)**	*Cluster*
Aguasmestas 1	4.8	2.2	10.9	63.6	18.5	689.5	Y
Allande 3	4.7	2.2	15.5	57.7	19.8	660.9	Y
Amandi	4.0	2.1	11.7	64.6	17.6	698.8	X
Andines 2	4.1	2.0	12.4	67.2	14.3	711.2	Y
Araujo*	4.1	2.5	11.2	64.5	17.8	695.9	X
Avellanosa*	4.0	2.4	12.4	65.6	15.6	702.2	X
Barreiros 1	4.0	1.9	11.6	66.9	15.6	711.2	Y
Barreiros 2	3.9	2.2	12.1	64.6	17.1	698.3	Y
Butler*	5.3	2.9	16.3	64.3	11.2	689.2	Z
Camponica*	4.1	2.2	11.3	66.0	16.4	704.9	Z
Casina	4.4	2.3	10.3	65.5	17.5	700.9	Z
Castrosin 2	4.6	2.2	13.4	62.9	16.9	687.0	Y
El peñueco 1	3.9	2.0	11.6	64.3	18.1	697.9	Z
Espinaredo	4.6	2.5	12.6	61.7	18.8	680.3	Y
Forcinas 1	4.1	2.1	11.2	66.6	15.9	708.3	X
Forcinas 2	4.1	2.4	14.2	63.9	15.4	693.8	Y
Grande*	4.2	2.3	14.1	61.7	17.7	682.0	X
Inclan	4.0	2.2	12.1	64.7	17.1	698.6	X
La riera 2	3.7	2.1	10.4	67.4	16.4	713.9	X
Las cuevas 1	4.2	2.1	10.8	66.3	16.5	706.3	Z
Llamazares 1	4.7	2.2	11.1	61.2	20.9	678.2	Y
Llamazares 2	3.7	2.0	10.1	66.8	17.4	711.5	X
Llanos 1	4.4	2.2	12.4	63.2	17.9	689.9	Y
Llanos 2	4.5	2.2	11.2	62.7	19.4	686.3	X
Morell*	3.9	2.1	12.7	65.0	16.4	700.7	X
Negret*	3.8	2.1	11.9	67.4	14.7	713.5	X
Pesoz 2	3.8	1.8	11.8	66.6	16.1	710.9	X
Priero 1	3.6	1.8	12.0	68.7	13.8	721.6	X
Priero 2	3.8	2.0	8.9	68.8	16.4	720.8	X
Pumares 1	4.2	2.3	14.3	61.6	17.6	682.1	Y
Pumares 2	4.4	2.1	11.1	63.2	19.1	690.0	Z
Pumares 4	4.3	2.3	11.6	63.4	18.4	690.3	X
Quirós	4.4	2.0	12.0	57.9	23.8	663.8	Y
Riocastiello 1	4.0	2.2	12.2	65.8	15.9	704.2	Y
Riocastiello 3	5.0	2.3	11.7	63.6	17.4	689.1	Z
Robriguero 2	4.2	2.3	13.3	63.2	17.0	689.8	Y
Rubiamo 1	4.0	2.1	11.7	64.3	17.9	697.2	X
San Pedro 1	4.1	2.1	12.4	64.1	17.3	695.2	X
San Pedro 3	4.4	2.3	12.8	64.0	16.6	693.2	Y
Santana 2	4.4	2.1	10.7	68.2	14.6	714.7	Z
Segorbe*	4.0	2.2	14.4	64.7	14.7	698.5	Y
Taranes 2	4.2	2.3	11.2	64.9	17.5	698.4	X
Tuñon 2	4.5	2.1	11.9	65.9	15.6	703.4	Z
Tuñon 3	4.0	2.2	11.5	65.4	17.0	702.3	X
Tuñon 4	4.3	2.1	11.2	64.5	17.9	696.7	Z
Yerbo 1	4.0	1.9	11.4	65.3	17.3	702.8	Y
Yerbo 2	4.3	2.1	11.2	66.0	16.4	704.0	Y

Supplementary Table 4. Fatty acid composition (%) of oils extracted from local and reference cultivars. *, non-local accessions.

**Accesions**	miristic	palmitic	plamitoleic	stearic	oleic	vaccenin c	linoleic	linolenic	arachidic	gondoic
Aguasmestas 1	0.02	7.51	0.17	2.61	75.71	1.48	12.35	0.05	0.06	0.05
Allande 3	0.02	5.51	0.08	2.25	77.31	1.28	13.41	0.04	0.06	0.05
Amandi	0.02	7.51	0.15	2.73	76.80	1.58	11.05	0.04	0.06	0.05
Andines 2	0.02	6.54	0.18	2.00	77.53	1.56	12.04	0.04	0.04	0.05
Araujo*	0.02	7.05	0.13	2.53	77.11	1.53	11.46	0.05	0.06	0.06
Avellanosa*	0.01	6.51	0.13	2.22	78.82	1.60	10.58	0.04	0.05	0.05
Barreiros 1	0.02	7.11	0.14	2.28	79.28	1.42	9.63	0.04	0.05	0.04
Barreiros 2	0.02	7.31	0.14	2.85	75.97	1.54	12.03	0.04	0.06	0.05
Butler*	0.03	7.52	0.15	2.63	78.41	1.39	9.70	0.05	0.06	0.06
Camponica*	0.02	6.94	0.15	2.41	81.59	1.46	7.22	0.05	0.08	0.08
Casina	0.03	7.75	0.19	2.27	77.09	1.47	11.04	0.04	0.06	0.05
Castrosin 2	0.02	7.17	0.14	3.29	75.65	1.28	12.27	0.04	0.08	0.05
El Peñueco 1	0.03	7.80	0.18	3.02	75.83	1.40	11.55	0.05	0.08	0.06
Espinaredo	0.02	6.61	0.13	2.32	73.55	1.58	15.65	0.04	0.05	0.05
Forcinas 1	0.02	7.10	0.15	2.05	75.71	1.75	13.11	0.03	0.04	0.05
Forcinas 2	0.02	6.11	0.10	2.09	78.13	1.42	11.97	0.05	0.05	0.05
Grande*	0.02	7.12	0.13	2.08	79.61	1.53	9.37	0.04	0.06	0.05
Inclan	0.02	6.83	0.14	2.53	76.42	1.57	12.35	0.04	0.06	0.05
La Riera 2	0.02	7.36	0.16	2.65	77.97	1.56	10.15	0.03	0.06	0.05
Las cuevas 1	0.03	6.84	0.21	2.36	78.03	1.54	10.79	0.05	0.07	0.08
Llamazares 1	0.02	7.50	0.15	2.84	75.65	1.38	12.29	0.05	0.07	0.05
Llamazares 2	0.02	7.78	0.18	2.54	76.73	1.56	11.06	0.03	0.05	0.04
Llanos 1	0.03	7.03	0.15	2.56	76.37	1.44	12.25	0.04	0.07	0.06
Llanos 2	0.02	7.07	0.16	2.16	73.70	1.73	15.04	0.04	0.04	0.05
Morell*	0.02	6.97	0.15	1.93	77.08	1.69	12.01	0.04	0.05	0.06
Negret*	0.02	7.34	0.18	2.35	74.19	1.61	14.17	0.04	0.05	0.05
Pesoz 2	0.03	7.41	0.18	2.13	77.70	1.61	10.78	0.04	0.05	0.06
Priero 1	0.02	6.35	0.13	2.06	79.29	1.51	10.49	0.03	0.05	0.06
Priero 2	0.01	7.58	0.18	2.22	78.83	1.77	9.29	0.03	0.04	0.05
Pumares 1	0.02	6.86	0.12	3.38	75.37	1.47	12.63	0.04	0.07	0.05
Pumares 2	0.03	7.50	0.17	2.21	77.50	1.53	10.91	0.05	0.06	0.05
Pumares 4	0.02	7.31	0.14	2.91	75.46	1.57	12.46	0.03	0.05	0.04
Quirós	0.03	6.93	0.16	3.02	75.13	1.46	13.07	0.06	0.08	0.07
Riocastiello 1	0.02	7.23	0.14	2.88	75.41	1.52	12.65	0.06	0.06	0.05
Riocastiello 3	0.03	7.85	0.16	2.94	78.70	1.31	8.83	0.05	0.07	0.06
Robriguero 2	0.02	6.54	0.11	3.00	79.34	1.34	9.49	0.05	0.07	0.05
Rubiamo 1	0.02	7.97	0.16	2.90	77.88	1.60	9.35	0.03	0.05	0.04
San Pedro 1	0.02	7.55	0.18	2.69	76.98	1.58	10.87	0.03	0.05	0.04
San Pedro 3	0.02	7.57	0.17	3.61	73.95	1.59	12.94	0.04	0.07	0.05
Santana 2	0.02	7.49	0.19	2.69	77.16	1.38	10.92	0.04	0.06	0.06
Segorbe*	0.02	6.74	0.17	2.43	78.43	1.53	10.51	0.05	0.06	0.06
Taranes 2	0.02	7.68	0.16	2.95	77.74	1.61	9.72	0.03	0.05	0.04
Tuñon 2	0.03	7.93	0.19	2.82	79.20	1.46	8.20	0.04	0.08	0.06
Tuñon 3	0.02	7.75	0.15	2.84	78.43	1.51	9.19	0.03	0.05	0.04
Tuñon 4	0.03	7.73	0.17	3.05	78.68	1.32	8.86	0.04	0.07	0.05
Yerbo 1	0.02	7.40	0.14	2.74	78.45	1.43	9.69	0.03	0.05	0.04
Yerbo 2	0.02	6.88	0.16	2.40	77.09	1.49	11.82	0.04	0.05	0.05

Supplementary Table 5 Squalene and tocopherol content in oils extracted from local and reference varieties (mg/kg). *, non local materials.

Accessions	squalene	α-tocopherol	β-tocopherol	γ-tocopherol	δ-tocopherol	Sum of tocopherols
Aguasmestas 1	246.8	415.0	14.2	31.4	3.3	463.9
Allande 3	260.9	515.9	20.0	26.1	1.3	563.2
Amandi	234.7	451.2	12.0	24.7	1.6	489.5
Andines 2	206.8	518.7	12.9	36.7	3.6	571.9
Araujo*	363.8	450.1	10.0	12.5	2.0	474.6
Avellanosa*	317.1	509.7	11.2	13.8	1.9	536.6
Barreiros 1	204.2	586.2	13.5	26.9	1.8	628.4
Barreiros 2	278.5	453.2	12.2	26.1	1.8	493.3
Butler*	393.4	465.2	12.5	28.9	3.1	509.6
Camponica*	300.1	372.4	7.7	33.5	3.2	416.8
Casina	612.3	532.6	13.3	27.0	3.4	576.4
Castrosin 2	337.6	553.3	16.7	31.2	4.5	605.7
El Peñueco 1	253.8	513.2	15.1	28.5	3.5	560.3
Espinaredo	248.3	579.3	17.0	31.0	2.1	629.5
Forcinas 1	456.0	354.2	7.7	13.8	1.3	377.1
Forcinas 2	279.0	578.4	12.7	17.5	2.5	611.1
Grande*	246.7	384.0	6.3	13.2	2.1	405.6
Inclan	277.3	437.2	12.2	15.0	1.5	466.0
La Riera 2	243.0	482.4	12.3	19.1	1.6	515.4
Las cuevas 1	335.5	462.1	11.5	24.5	1.2	499.3
Llamazares 1	282.0	432.5	15.4	23.3	3.5	474.7
Llamazares 2	295.5	533.4	12.7	27.1	2.2	575.3
Llanos 1	283.2	533.3	13.8	28.2	3.2	578.5
Llanos 2	550.6	340.8	12.4	23.0	2.6	378.8
Morell*	309.7	461.3	13.1	17.2	1.3	492.9
Negret*	267.7	490.2	11.3	21.4	3.4	526.3
Pesoz 2	293.0	397.5	10.0	22.4	1.2	431.1
Priero 1	433.0	364.7	8.0	17.3	2.3	392.3
Priero 2	586.6	319.4	6.8	5.9	0.7	332.8
Pumares 1	225.6	404.0	11.7	18.7	1.8	436.2
Pumares 2	255.5	459.2	15.1	29.7	7.4	511.4
Pumares 4	325.2	435.0	12.4	19.9	2.8	470.1
Quirós	232.6	444.1	20.8	14.7	4.3	483.8
Riocastiello 1	265.5	500.3	12.5	25.7	1.7	540.2
Riocastiello 3	269.6	520.9	16.9	30.0	5.3	573.1
Robriguero 2	204.7	515.2	13.6	34.7	1.5	565.0
Rubiamo 1	225.4	348.1	8.9	16.2	2.5	375.7
San Pedro 1	210.7	544.7	13.5	22.9	1.6	582.7
San Pedro 3	266.7	424.9	14.5	20.5	3.1	463.0
Santana 2	199.0	505.2	15.6	28.8	4.0	553.7
Segorbe*	216.8	669.5	16.6	18.3	1.9	706.4
Taranes 2	333.0	367.1	11.0	15.9	3.5	397.5
Tuñon 2	259.2	481.3	14.7	26.2	4.8	526.9
Tuñon 3	207.2	471.1	14.8	22.7	4.7	513.3
Tuñon 4	221.4	557.1	17.9	32.4	5.5	612.9
Yerbo 1	223.4	581.9	15.4	27.2	2.2	626.6
Yerbo 2	356.0	545.7	16.1	32.3	4.8	598.8
